# Supplementary material for: Spin-orbit quantum impurity in a topological magnet
Source: Nat Commun. 2020 Sep 4;11:4415. doi: 10.1038/s41467-020-18111-6 (PMC7474094; doi:10.1038/s41467-020-18111-6)
Supplement: Supplementary file 1 — Supplementary Information [file 41467_2020_18111_MOESM1_ESM.pdf]

Supplementary Information

Spin-orbit quantum impurity in a topological magnet

Jia-Xin Yin, Nana Shumiya, Yuxiao Jiang, Huibin Zhou, Gennevieve Macam, Hano Omar Mohammad Sura et al.

Supplementary Note 1

Additional first-principles calculations. To understand the local In-Co coupling, we calculate the charge densities of different systems as shown in Fig. 1. Figure 1a shows the charge density difference (Δρ) between an In-doped $Co_3Sn_2S_2$ system (ρ(In-CSS)), an isolated In atom (ρ(In)), and a $Co_3Sn_2S_2$ system with a Sn vacancy (ρ(CSS-Sn vac)). The calculation shows that there is local charge coupling between the In atom and the Co kagome lattice atoms. Figure 1b shows another charge density difference (Δρ') between an In-doped $Co_3Sn_2S_2$ system (ρ(In-CSS)), and a $Co_3Sn_2S_2$ system (ρ(CSS)). This calculation shows that the additional charge variation induced by the In atom as a substitutional impurity also couples with the Co kagome lattice. Both calculations demonstrate the local In-Co charge coupling.

The local charge In-Co charge coupling also has an impact on the local magnetism as shown in Fig. 2. The local magnetic moment for each atom is calculated through the sum of all occupied spin-up and spin-down local density of states. The calculated magnetic structure of an In-doped $Co_3Sn_2S_2$ system (Fig. 2a) shows that the local moment mainly arises from the Co atoms in the kagome lattice, which reduces a little for the three Co atoms near the In impurity. Figure 2b shows more details of the calculated magnetic structure for the top In-Sn, S and Co layers. The moment of In impurity is $-0.026\mu_B$, which is an order of magnitude smaller than that of Co ($\sim+0.3\mu_B$), and of similar value as that of Sn lattice atom ($\sim-0.02\mu_B$ at far away positions or in the bulk). Since the magnetism mainly arises from the Co atom in the kagome lattice and no additional large local magnetic moment is directly introduced by the In impurity, it is more reasonable to treat the In atom as a nonmagnetic impurity in this system for the theoretical modeling.

We further perform a first-principles calculation of the triplet impurity case. The supercell consists of a periodically repeating 3×3-slab with a thickness of a bulk unit cell and a vacuum space of ∼14 Å along the *z*-direction. The slab is cleaved to reveal the Sn-terminating surface and three surface Sn atoms are replaced with In to simulate the In trimer impurity. The energy cutoff was set at 300 eV and the energies in self-consistent calculations were converged until 10^{-4} eV. The Brillouin zone was sampled using a Gamma-centered 3×3×1 Monkhorst-Pack grid. Without spin-orbit coupling, each In impurity exhibits two sharp spin-down polarized bound states in the local density of states calculation (Fig. 3a). The antibonding state σ* is much stronger than bond state σ, consistent with the symmetry analysis that σ* is doubly degenerate. With including spin-orbit coupling, σ* is splits into two peaks, and there are totally three impurity states (Fig. 3b), consistent with the experimental observation.

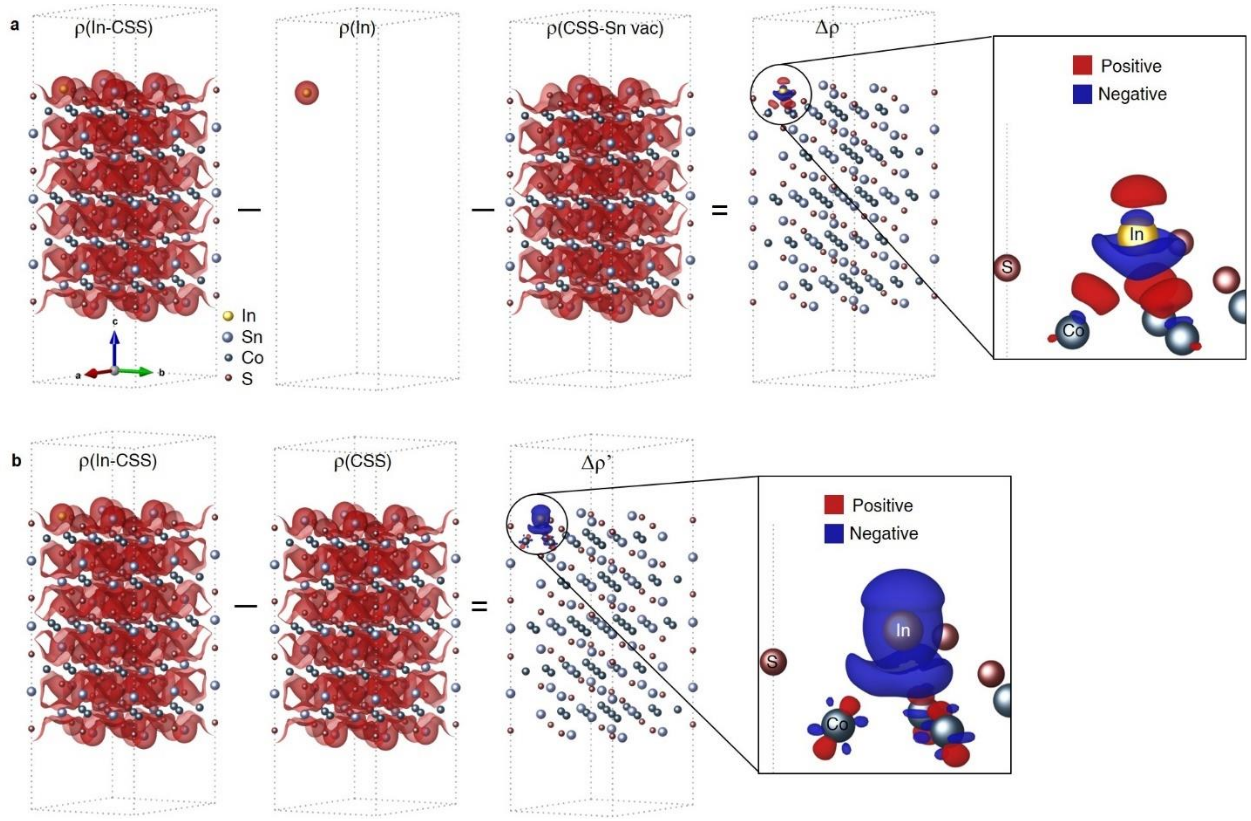

Supplementary Figure 1: Local In-Co charge coupling. a Charge density difference (Δρ) between an In-doped $Co_3Sn_2S_2$ system (ρ(In-CSS)), an isolated In atom (ρ(In)), and a $Co_3Sn_2S_2$ system with a Sn vacancy (ρ(CSS-Sn vac)). **b** Charge density difference (Δρ') between an In-doped $Co_3Sn_2S_2$ system (ρ(In-CSS)), and a $Co_3Sn_2S_2$ system (ρ(CSS)). The isosurface levels of the individual and differential systems are 8×10^{-06} eÅ^{-3} and 8×10^{-07} eÅ^{-3}, respectively. The spin-orbit coupling is considered in this calculation.

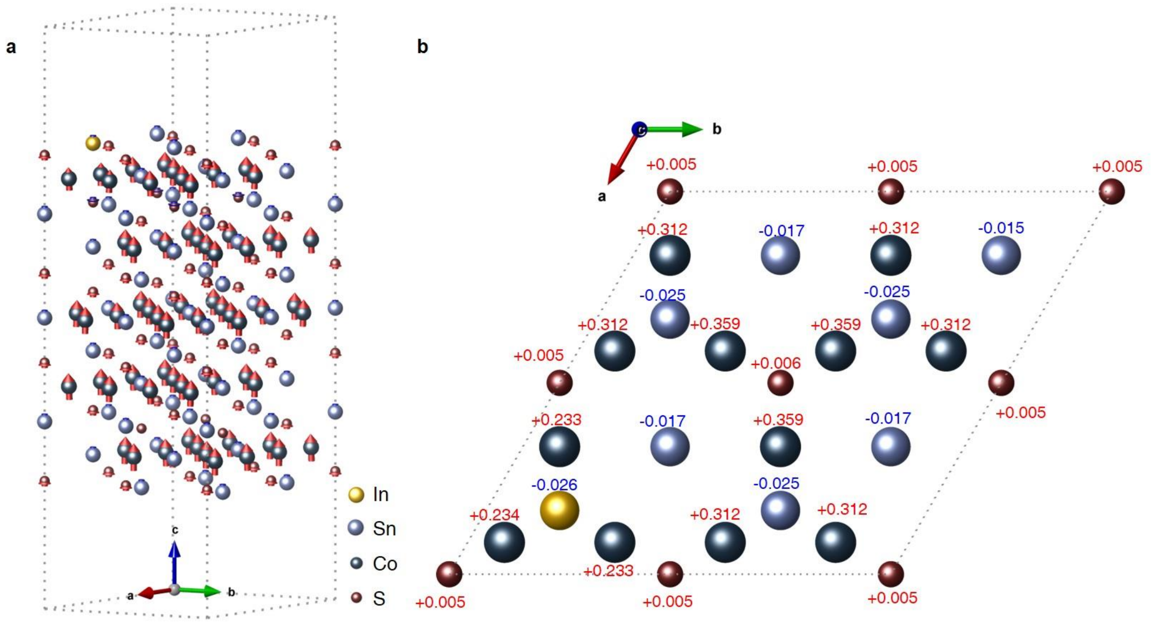

Supplementary Figure 2: Impact of In impurity on local magnetism. a Magnetic structure of the In-doped $Co_3Sn_2S_2$ system. The arrows in the atoms are proportional to the magnetic moment size. **b** Magnetic structure for the top In-Sn, S and Co layers. The magnetic moment is marked for each atom with the units of μ_B. The spin-orbit coupling is considered in this calculation.

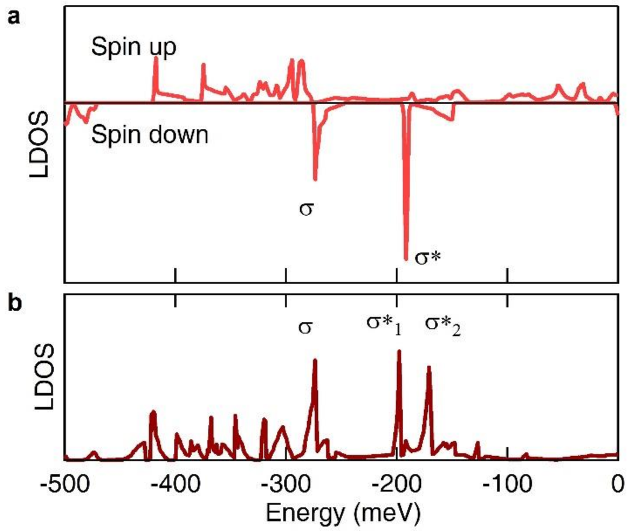

Supplementary Figure 3: Impact of spin-orbit coupling on triple impurity state. a, First-principles calculation of the spin-resolved local density of states at the In triple impurity without spin-orbit coupling. **b,** First-principles calculation of the local density of states at the In triple impurity with spin-orbit coupling.

Supplementary Note 2

A heuristic model for the magnetic impurity resonance We show that nonmagnetic scattering in the spin-orbit coupled magnetic system can generate magnetic impurity resonance, by considering a nearest-neighbor hopping (t) model on the kagome lattice, with spin-orbital coupling (λ) and an overall Zeeman field (B) within the T-matrix approach. We assume that the relevant bands stem from a single orbital (d_{z^2}) at each Co-atom in the kagome lattice[13], and the In-Co coupling effectively introduces local potential scattering on the kagome lattice. The unit cell of the kagome lattice includes three Co-atoms. The lattice vectors are $\boldsymbol{a_1} = a(1,0), \boldsymbol{a_2} = a(1,\sqrt{3}), \boldsymbol{a_3} = \boldsymbol{a_2} - \boldsymbol{a_1}$. For the calculation of the impurity-free Green function, we identify the $\boldsymbol{k}$-vectors within the Brillouin zone for a certain lattice size. We assume that there are N=100 lattice points along the $\mathbf{a}_1$ and $\mathbf{a}_2$ primitive lattice vectors. The model we will utilize to represent the clean system is:

$$H = H_{Kin} + H_{SOC} + H_B \tag{1}$$

with

$$H_{Kin}(\boldsymbol{k}) = 2t \begin{pmatrix} 0 & \cos \boldsymbol{k}\cdot\boldsymbol{a_1} & \cos \boldsymbol{k}\cdot\boldsymbol{a_2} \\ \cos \boldsymbol{k}\cdot\boldsymbol{a_1} & 0 & \cos \boldsymbol{k}\cdot\boldsymbol{a_3} \\ \cos \boldsymbol{k}\cdot\boldsymbol{a_2} & \cos \boldsymbol{k}\cdot\boldsymbol{a_3} & 0 \end{pmatrix} \tag{2}$$

$$H_{SOC}(\boldsymbol{k}) = 2\lambda i \begin{pmatrix} 0 & \cos \boldsymbol{k}\cdot(\boldsymbol{a_2}+\boldsymbol{a_3}) & -\cos \boldsymbol{k}\cdot(\boldsymbol{a_3}-\boldsymbol{a_1}) \\ -\cos \boldsymbol{k}\cdot(\boldsymbol{a_2}+\boldsymbol{a_3}) & 0 & \cos \boldsymbol{k}\cdot(\boldsymbol{a_1}+\boldsymbol{a_2}) \\ \cos \boldsymbol{k}\cdot(\boldsymbol{a_3}-\boldsymbol{a_1}) & -\cos \boldsymbol{k}\cdot(\boldsymbol{a_1}+\boldsymbol{a_2}) & 0 \end{pmatrix} \tag{3}$$

$$H_B(k) = B \begin{pmatrix} 1 & 0 & 0 \\ 0 & 1 & 0 \\ 0 & 0 & 1 \end{pmatrix} \otimes \sigma_z \tag{4}$$

where the 3×3 structure is due to the Co-sites within the unit cell, σ_i are Pauli-matrices within the spin-space of the states.

Since the overall Hamiltonian is diagonal in spin space, we can and will consider each spin separately. The free Green function matrix can then be shown to be:

$$\underline{\underline{G_0^\sigma}}(k,\omega) = \sum_\nu \frac{\widehat{P_\nu}(k)}{\omega - E_\nu^\sigma(k) + i\eta} = [\omega - H^\sigma(k) + i\eta]^{-1} \tag{5}$$

where $\widehat{P_\nu}$ is the projection operator onto the Hamiltonian eigenstate ν, and the real space Green function is:

$$\underline{\underline{G_0^\sigma}}(\boldsymbol{k},\omega) = \sum_\nu \frac{\widehat{P_\nu}(\boldsymbol{k})}{\omega - E_\nu^\sigma(\boldsymbol{k}) + i\eta} = [\omega - H^\sigma(\boldsymbol{k}) + i\eta]^{-1} \tag{6}$$

where $\widehat{P_\nu}$ is the projection operator onto the Hamiltonian eigenstate ν, and the real space Green function is:

$$\underline{\underline{G_0^\sigma}}(\boldsymbol{r_i} - \boldsymbol{r_j},\omega) = \frac{1}{V}\sum_\nu [\omega - H^\sigma(k) + i\eta]^{-1} \exp\,[i\boldsymbol{k}\cdot(\boldsymbol{r_i} - \boldsymbol{r_j})] \tag{7}$$

We are interested in the full, on-site Green function in the presence of an impurity at $\mathbf{r}$ = (0,0). The Dyson equation of the general, real-space Green function is:

$$\underline{\underline{G^\sigma}}(\boldsymbol{r_i}, \boldsymbol{r_j}, \omega) = \underline{\underline{G_0^\sigma}}(\boldsymbol{r_i} - \boldsymbol{r_j}, \omega) + \underline{\underline{G_0^\sigma}}(\boldsymbol{r_i}, \omega) + \underline{\underline{V}}\ \underline{\underline{G^\sigma}}(0, \boldsymbol{r_j}, \omega) \tag{8}$$

where $\underline{\underline{V}}$ is the contribution to the Hamiltonian from the impurity, which we assume to be nonmagnetic (V_{mag} = 0 and only considering the potential scattering V_{Pot}):

$$\underline{\underline{V}} = V_{Pot}\begin{pmatrix} 1 & 0 & 0 \\ 0 & 1 & 0 \\ 0 & 0 & 1 \end{pmatrix} \otimes \sigma_0 + V_{Mag}\begin{pmatrix} 1 & 0 & 0 \\ 0 & 1 & 0 \\ 0 & 0 & 1 \end{pmatrix} \otimes \sigma_z \tag{9}$$

where we included the magnetic term for generality. Crucially now:

$$\underline{\underline{G^\sigma}}(0, \boldsymbol{r_j}, \omega) = \underline{\underline{G_0^\sigma}}(-\boldsymbol{r_j}, \omega) + \underline{\underline{G_0^\sigma}}(0, \omega)\underline{\underline{V}}\ \underline{\underline{G^\sigma}}(0, \boldsymbol{r_j}, \omega) = \underline{\underline{G_0^\sigma}}(\boldsymbol{r_j}, \omega) + \underline{\underline{G_0^\sigma}}(0, \omega)\underline{\underline{V}}\ \underline{\underline{G^\sigma}}(0, \boldsymbol{r_j}, \omega)$$

$$\Rightarrow \underline{\underline{G^\sigma}}(0, \boldsymbol{r_j}, \omega) = [1 - \underline{\underline{G_0^\sigma}}(0, \omega)\underline{\underline{V}}]^{-1}\underline{\underline{G_0^\sigma}}(\boldsymbol{r_j}, \omega) \tag{10}$$

and thus

$$\underline{\underline{G^\sigma}}(\boldsymbol{r_i}, \boldsymbol{r_j}, \omega) = \underline{\underline{G_0^\sigma}}(\boldsymbol{r_i} - \boldsymbol{r_j}, \omega) + \underline{\underline{G_0^\sigma}}(\boldsymbol{r_i}, \omega)\underline{\underline{V}}[1 - \underline{\underline{G_0^\sigma}}(0, \omega)\underline{\underline{V}}]^{-1}\underline{\underline{G_0^\sigma}}(\boldsymbol{r_j}, \omega)$$

$$\equiv \underline{\underline{G_0^\sigma}}(\boldsymbol{r_i} - \boldsymbol{r_j}, \omega) + \underline{\underline{G_0^\sigma}}(\boldsymbol{r_i}, \omega)\underline{\underline{T^\sigma}}(\omega)\underline{\underline{G_0^\sigma}}(\boldsymbol{r_i}, \omega) \tag{11}$$

From this we finally have an expression for the on-site Green function:

$$\underline{\underline{G^\sigma}}(\boldsymbol{r_i}, \boldsymbol{r_j}, \omega) = \underline{\underline{G_0^\sigma}}(0, \omega) + \underline{\underline{G_0^\sigma}}(\boldsymbol{r_i}, \omega)\underline{\underline{T^\sigma}}(\omega)\underline{\underline{G_0^\sigma}}(\boldsymbol{r_i}, \omega) \tag{12}$$

At this point, we must calculate the T-matrix and the impurity-free Green function, which we will do numerically.

We set the lattice constant *a* of the kagome lattice to be 1, while the hopping integral, *t* = 1. Thus length is measured in *a*, while energy is measured in *t*. Figure 4a shows the dispersion along the K−Γ−K direction of the Brillouin zone calculated directly from diagonalizing $H(\boldsymbol{k})$, which captures the key band features in the first-principle calculation in Ref. 11. We now turn to the DOS from the full Green function. Figure 4b shows the spin-resolved LDOS calculated from the impurity-free Green function and the full Green function at the impurity site, *r* = (0,0), for an impurity potential of V_{Pot} = 5. A spin-down DOS peak is found in the band gap between spin up and spin down. The spin-up resonance may be damped by coupling with other bands, and more realistic band structures need to be considered to fully produce the experimental results, which deserves further model investigation. This minimum model calculation supports that the nonmagnetic scattering in this magnetic kagome lattice system can generate strong magnetic resonance.

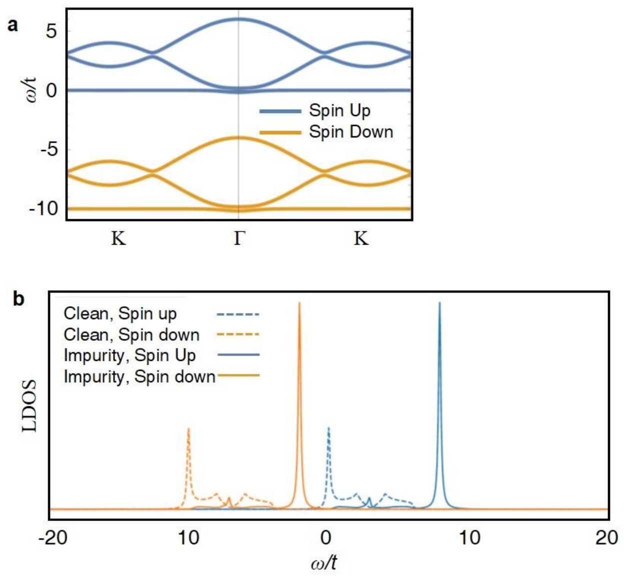

Supplementary Figure 4. Nonmagnetic scattering induced magnetic resonance. a, Calculated band structure along the high symmetry direction with parameters: t = 1, μ = 3, λ = 0.05, and B = 5. **b,** The spin-resolved LDOS in the clean case and at the impurity site with V_{Pot} = 5.

Supplementary Note 3

Fundamental tight-binding model for impurity state coupling. We consider a tight-binding model and construct an effective Hamiltonian for coupled impurities:

$$H = H_k + H_{SOC} + H_Z \tag{13}$$

Here H_k represents the hopping term:

$$H_k = \sum_{ij} t c_i^\dagger c_j \tag{14}$$

where t is inter-impurity hopping amplitude and $c_i(c_j^\dagger)$ is the electron annihilation(creation) operator in the spinor notation at impurity site i(j). H_{SOC} is the spin-orbit interaction:

$$H_{SOC} = i \sum_{ij} \lambda \nu_{ij} (c_i^\dagger \sigma_z c_j) \tag{15}$$

where λ is spin-orbit coupling amplitude, σ_z is the spin Pauli matrix and $\nu_{ij} = (\boldsymbol{d}_i \times \boldsymbol{d}_j) \cdot \boldsymbol{z}$ with $\boldsymbol{d_i}$ and $\boldsymbol{d}_j$ denoting the unit vectors along the two bonds that the electron traverses from site i to site j. H_Z is the Zeeman term induced by out-of-plane magnetization of the material:

$$H_Z = \sum_i B c_i^\dagger \sigma_z c_i \tag{16}$$

where B is the effective Zeeman field. We first consider double impurity case, the Hamiltonian reads:

$$H_{double} = \begin{pmatrix} 0 & t \\ t & 0 \end{pmatrix} \otimes I + BI \otimes \sigma_z \tag{17}$$

The spin-orbit coupling does not play a role in the double impurity case. We assume the effective Zeeman field polarizes the impurity bound state such that two spin channels are well separated from each other. For each spin channel, we obtain a bonding state with energy *t* and an anti-bonding state with energy -*t*.

For the triple impurity case, the spin-orbit coupling effect has to be considered. The Hamiltonian in real space is:

$$H = \begin{pmatrix} 0 & t & t \\ t & 0 & t \\ t & t & 0 \end{pmatrix} \otimes I + \begin{pmatrix} 0 & i\lambda & -i\lambda \\ -i\lambda & 0 & i\lambda \\ i\lambda & -i\lambda & 0 \end{pmatrix} \otimes \sigma_z + BI \otimes \sigma_z \tag{18}$$

Considering only one spin channel, we get three energy levels: $2t, -t \pm \sqrt{3}\lambda$. Without spin-orbit coupling, the system has one single state with energy $2t$ and two degenerate eigenstates with energy $-t$. This degeneracy is protected by C_{3v} symmetry. The spin-orbit coupling breaks the mirror symmetry and lifts the two degenerate levels with energy difference $2\sqrt{3}\lambda$.

From this basic coupled-impurity model, the bound state energies for single, doubly, and triply coupled impurities will be (0), (*t*, -*t*), (2*t*, -*t*-$\sqrt{3}\lambda$, -*t*+$\sqrt{3}\lambda$), respectively. Additional local hole doping effects need to be considered to fully explain the observed states (A), (B, C), and (D, E, F) in Fig. 3b, which could be related to the fact that In is a hole dopant in Sn layer[20-22]. Remarkably, the key ratio (D-(E+F)/2)/(B-C) = (310-(220+170)/2)/(315-240) = 1.5 ± 0.1, which is the expected value *3t/2t*=1.5, confirming the internal consistency of our experiment with respect to this basic coupled impurity model.
